# Low 2-Dimensional CD4 T Cell Receptor Affinity for Myelin Sets in Motion Delayed Response Kinetics

**DOI:** 10.1371/journal.pone.0032562

**Published:** 2012-03-07

**Authors:** Kristen M. Rosenthal, Lindsay J. Edwards, Joseph J. Sabatino, Jennifer D. Hood, Heather A. Wasserman, Cheng Zhu, Brian D. Evavold

**Affiliations:** 1 Department of Microbiology and Immunology, Emory University, Atlanta, Georgia, United States of America; 2 Coulter Department of Biomedical Engineering, Georgia Institute of Technology, Atlanta, Georgia, United States of America; University Paris Sud, France

## Abstract

T cells recognizing self-peptides that mediate autoimmune disease and those that are responsible for efficacious immunity against pathogens may differ in affinity for antigen due to central and peripheral tolerance mechanisms. Here we utilize prototypical self-reactive (myelin) and viral-specific (LCMV) T cells from T cell receptor (TCR) transgenic mice (2D2 and SMARTA, respectively) to explore affinity differences. The T cells responsive to virus possessed >10,000 fold higher 2D affinity as compared to the self-reactive T cells. Despite their dramatically lower affinity for their cognate ligand, 2D2 T cells respond with complete, albeit delayed, activation (proliferation and cytokine production). SMARTA activation occurs rapidly, achieving peak phosphorylation of p38 (1 minute), Erk (30 minutes), and Jun (3 hours) as well as CD69 and CD25 upregulation (3 and 6 hours, respectively), with a corresponding early initiation of proliferation. 2D2 stimulation with MOG results in altered signaling – no phospho-Erk or phospho-p38 accumulation, significantly delayed activation kinetics of Jun (12 hours), and delayed but sustained SHP-1 activity – as well as delayed CD69 and CD25 expression (12–24 hours), and slow initiation of proliferation. This delay was not intrinsic to the 2D2 T cells, as a more potent antigen with >100-fold increased 2D affinity restored rapid response kinetics in line with those identified for the viral antigen. Taken together, these data demonstrate that time can offset low TCR affinity to attain full activation and suggest a mechanism by which low affinity T cells participate in autoimmune disease.

## Introduction

Every T cell must be able to differentiate between high and low potency ligands to generate the appropriate response after T cell receptor (TCR) binding [1]–[3], as antigens vary in their capacity to stimulate a given T cell. The library of peptides for a specific TCR includes agonists, partial or weak agonists, and antagonists [4]. In the case of CD8+ OT-I T cells, these ligands can span a >1,000 fold range in effective 2D affinity [5]. The TCR can thus integrate the strength of ligand binding and impart the appropriate response, ranging from full activation to anergy to antagonism [6], [7].

Generally, self-reactive T cells mediating autoimmune disease are thought to be lower affinity than pathogen-specific T cells due to various tolerance mechanisms [8], [9]. Recently, we utilized a micropipette-based assay (where both the TCR and pMHC are membrane-bound) to assess the effective 2D affinity of T cells during various immune responses [10]. This report identified participation of lower affinity T cells in both pathogenic and autoimmune responses, although stimulation with a myelin-specific self-peptide important in murine EAE induction (MOG_35–55_) results in a greater frequency of tetramer negative low affinity T cells as compared to a pathogen-derived epitope in murine LCMV (gp_61–80_) [10].

When shaping an immune response, both the TCR affinity and duration of antigen encounter play roles in directing the outcome of T cell activation. During an acute infection, the presence of foreign antigen is transient and allows for robust T cell expansion followed by contraction to the memory state as pathogen is cleared [11]. During chronic infections, pathogenic antigens can be present for an extended time, which can lead to deletion or exhaustion of the T cells [12]–[14]. Self-peptide antigens are constantly produced and presented [15], [16], yet intriguingly, T cells that propagate autoimmune disease can seemingly avoid exhaustion or regulation.

As a first step to understand the apparent differences in T cell activation, we utilized LCMV specific (SMARTA) [17], [18] and myelin-reactive (2D2) [19] CD4^+^ TCR transgenic mouse models and identified a >10,000 fold lower effective 2D affinity in 2D2 T cells that resulted in a substantial decrease in functional sensitivity to myelin and a complete absence of peptide:MHC class II tetramer reactivity. In spite of this dramatically decreased affinity for cognate ligand, 2D2 T cells successfully proliferated and produced cytokines, although with a temporal delay in the T cell response that manifested as an absence of detectable phosphorylated Erk and p38 and significantly delayed activation kinetics of SHP-1, Jun, CD69, and CD25. In contrast, activation occurred rapidly in the pathogen specific T cells. The delay in response to myelin was not intrinsic to the self-reactive T cells, as the 2D2 response to a more potent antigen with a >100-fold increase in effective 2D affinity gave rapid response kinetics. Moreover, the low-affinity MOG peptide must be displayed for a protracted time to initiate a robust response. Our data demonstrate that extended time of antigen presentation can compensate for lower TCR affinity for self and allow for accumulation of signals and eventual full activation of CD4^+^ T cells.

## Results

### The relative 2D affinity of CD4^+^ T cells

**Figure 1:**
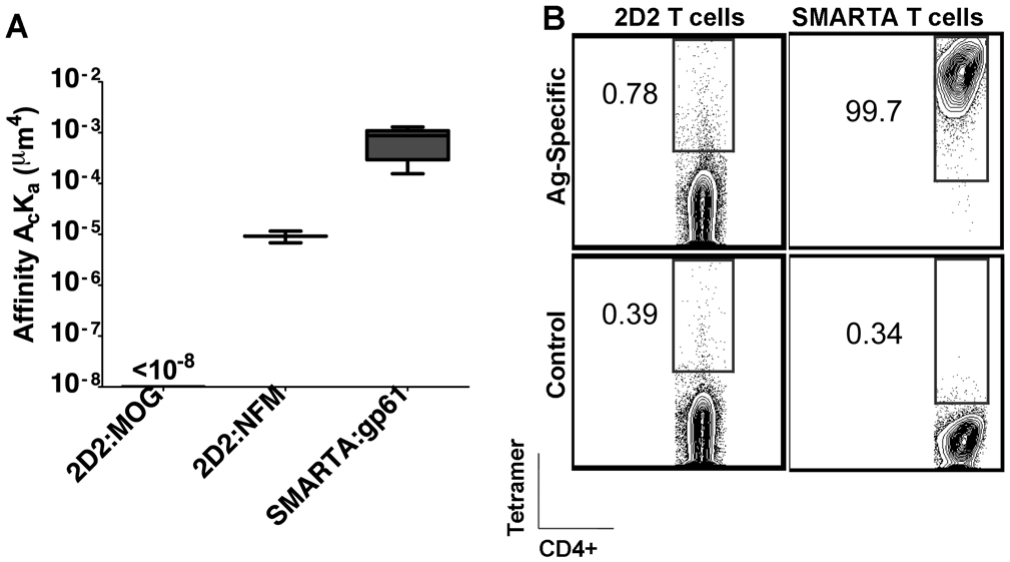
The effective 2D affinity of 2D2 and SMARTA CD4^+^ T cells differs. (A) 2D2 or SMARTA T cells were stained with antigen-specific I-A^b^ tetramer (MOG_38–49_ or gp_66–77_, respectively) or a negative control I-A^b^ tetramer and analyzed by flow cytometry, gated on CD4^+^ cells. (B) Human RBCs were coated with the indicated pMHC complex and brought into contact with the corresponding T cell by micropipette numerous times. The resulting adhesion frequency was used to derive the effective 2D affinity (*A_c_K_a_*, in µm^4^). All experiments were performed at least three times.

Using a micropipette-based binding assay the effective two-dimensional (2D) affinity, which measures receptor∶ligand binding in the context of a cell membrane, can define differences in affinity of TCR:pMHC with greater resolution than other measurements [5], [10]. In this assay, a single T cell is brought in and out of contact with a red blood cell (RBC) coated with pMHC class II monomers to yield an adhesion probability (the percentage of adhesions out of the total number of contacts). The adhesion probability allows for derivation of the effective 2D affinity of the TCR:pMHC [5], [20], [21]. SMARTA TCR interacts with gp_66–77_:I-A^b^ with high affinity (Figure 1a). In contrast, adhesion of 2D2 CD4^+^ T cells to RBCs coated with >2000 molecules/um^2^ MOG_38–49_:I-A^b^ was still negligible, indicating a <10^−8^ µm^4^ affinity (Figure 1a). It has recently been shown that, in addition to MOG, 2D2 T cells can respond better to a CNS self-epitope from the neurofilament medium chain (NFM) [22]. NFM_15–35_ contains 6 out of 9 identical amino acid residues to the core epitope of MOG_35–55_ and conserves all of the recognized TCR contact residues [22]. In contrast to MOG_35–55_, the adhesion between 2D2 TCR and NFM_18–30_:I-A^b^ is measurable in the micropipette assay and results in an affinity approximately 80-fold lower than that of SMARTA T cells (9.22×10^−6^ µm^4^ compared to 7.32×10^−4^ µm^4^) (Figure 1a).

Another gauge of TCR affinity uses the extent of peptide:MHC II tetramer staining detected by flow cytometry [23]–[25]. Previously, pMHC II tetramers were shown to detect MOG_35–55_ CD4^+^ T cells in EAE [10], [26], [27], but on detailed analysis, MOG:I-A^b^ tetramer failed to identify most of the MOG-specific T cells [10]. In accordance with the undetectable 2D affinity of MOG:I-A^b^ interaction with 2D2 TCR, there was a lack of MOG:I-A^b^ tetramer staining in 2D2 T cells, whereas all of the SMARTA T cells were positive for tetramer staining (Figure 1b).

Peptides, particularly longer peptides, may bind to MHC class II molecules in multiple 9-mer epitopes, or registers [28]–[32]. To define the 2D2 T cells' response to the core epitope engineered into the MOG_38–49_:I-A^b^ monomer, we assessed the proliferative capacity of 2D2 T cells to a panel of overlapping, truncated peptides from the MOG_35–55_ sequence (Table I). This assay revealed that the core epitope for 2D2 T cells is MOG_39–48_, which is the basis of the MOG:I-A^b^ construct (Table I), and demonstrated that the absence of MOG:I-A^b^ reactivity in tetramers or by 2D micropipette analysis was not due to the register of the antigenic epitope.

**Table 1:** The 2D2 core MOG epitope is MOG_39–48_.

MOG Epitope	35	36	37	38	39	40	41	42	43	44	45	46	47	48	49	50	51	52	53	54	55	Proliferative Response
35–55	M	E	V	G	W	Y	R	S	P	F	S	R	V	V	H	L	Y	R	N	G	K	+++
37–50			V	G	W	Y	R	S	P	F	S	R	V	V	H	L						+++
37–46			V	G	W	Y	R	S	P	F	S	R										−
38–47				G	W	Y	R	S	P	F	S	R	V									+
39–48					W	Y	R	S	P	F	S	R	V	V								+++
40–49						Y	R	S	P	F	S	R	V	V	H							−
41–50							R	S	P	F	S	R	V	V	H	L						−
41–55							R	S	P	F	S	R	V	V	H	L	Y	R	N	G	K	−
42–50								S	P	F	S	R	V	V	H	L						−
44–54										F	S	R	V	V	H	L	Y	R	N	G		−

Splenocytes were harvested and dose response curves were generated with the indicated peptides up to a maximal concentration of 100 µM to determine the proliferative capacity of 2D2 CD4^+^ T cells. The nested sets of peptides were generated from the known full length MOG_35–55_ epitope. Three pluses represent proliferation similar to the parent epitope, with each deduction of a plus representing a log shift in the dose required for maximal proliferation. A minus represents no proliferation above background.

### Functional avidity of CD4^+^ T cells

**Figure 2:**
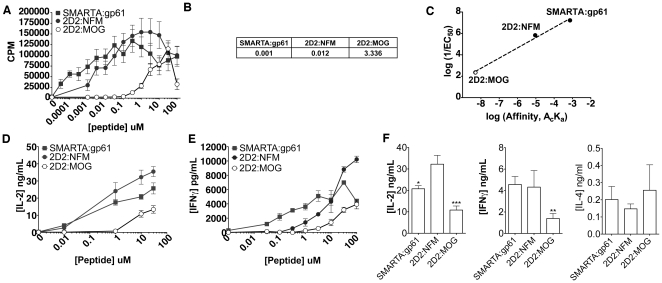
2D2 splenocytes stimulated with MOG have a low functional avidity. (A) 6×10^5^ splenocytes from SMARTA or 2D2 mice were stimulated with various doses of the indicated antigen for 72 hrs. and ^3^H-Thy was added during the last 18 hrs. to assess proliferation. (B) The concentration of peptide needed to reach the half-maximal response (EC_50_ values, µM) were derived from the above proliferation assay using GraphPad Prism. (C) The reciprocal EC_50_ was plotted against the effective 2D affinity. The open circle for 2D2:MOG denotes the uncertainty of 2D2:MOG affinity, as it was below 10^−8^ µm^4^, the limit of detection for this assay. (D) 1.5×10^6^ splenocytes were stimulated for 24 hrs. and supernatants were harvested to determine the amount of IL-2 by ELISA. (E) 1.5×10^6^ splenocytes were stimulated for 24 hrs. and supernatants were harvested to determine the amount of IFN- γ by ELISA. (F) 1.5×10^6^ splenocytes were stimulated with 10 µM of the indicated antigen and supernatants were harvested (24 hrs. for IL-2 and IFN-γ or 48 hrs. for IL-4) to determine the amount of cytokine by ELISA (*P* value: * = 0.025, ** = 0.005, ***<0.002). All experiments were performed at least three times.

T cell functional avidity, defined by the amount of antigen needed for half-maximal response, is often used as a surrogate of TCR affinity for expressing the potency of an antigen [33]–[36]. SMARTA splenocytes showed some proliferation at the lowest dose of gp61 tested (30 pM) while 2D2 splenocytes did not respond until much higher doses of MOG were reached (0.3–1 µM) (Figure 2a). Indeed, the EC_50_ of 2D2 T cells for MOG was more than 3,000 times higher than that of SMARTA T cells (3.3 µM compared to 0.001 µM) (Figure 2b). Although MOG:2D2 binding is too weak to be detected by either flow cytometry or 2D micropipette analysis, stimulation with this self-peptide still resulted in activation of 2D2 T cells. 2D2 cells responded to lower doses of NFM (starting at 1 nM) and by EC_50_ required approximately 10-fold more peptide than that of SMARTA cells (∼0.01 vs 0.001 µM) and 100-fold less peptide than that of 2D2 cells stimulated with MOG (∼3 µM) (Figure 2a,c). Additionally, antigen-dependent cytokine production, assessed by IL-2 production, paralleled the proliferative capacity of the cells (Figure 2d). A similar trend was observed with IFN-γ –in that 2D2:MOG stimulation resulted in less cytokine production than either SMARTA:gp61 or 2D2:NFM across the range of antigen concentrations (Figure 2e). Some have previously reported that low affinity T cell stimulation can result in cytokine skewing to a Th2 response [37]; however further cytokine analysis revealed 2D2:MOG produced lower amounts of IL-2 and IFN-γ than either SMARTA:gp61 or 2D2:NFM (*P* = 0.0018 and *P* = 0.005, respectively) with only minimal levels of IL-4 produced regardless of the antigen (Figure 2f). Taken together, this suggests that although 2D2:MOG results in less cytokine production, there is no evidence of phenotypic skewing.

Thus, 2D2 T cells have a relatively high functional avidity for NFM, with proliferation and IL-2 production closer to the corresponding functional avidity of SMARTA cells than to 2D2 cells for MOG (Figure 2). The hierarchy of the ligands shows that SMARTA:gp61, a TCR:foreign antigen interaction, is the most potent, followed by 2D2:NFM and, finally, 2D2:MOG. Overall, the effective 2D affinities of these CD4^+^ T cell clones for their ligand correlates to some extent with their functional avidity (Figure 2c).

### Altered signaling events in low affinity CD4^+^ T cells

**Figure 3:**
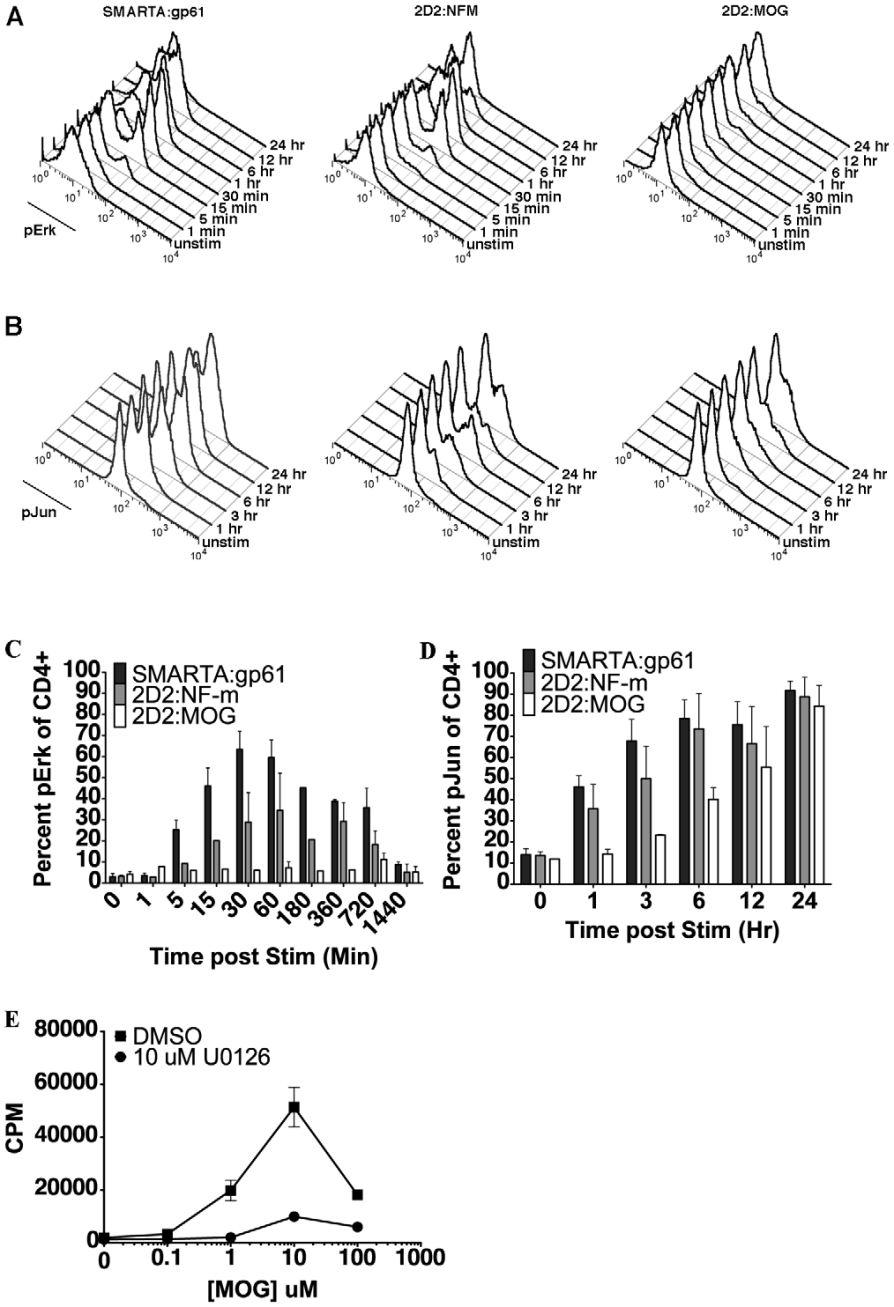
2D2 cells stimulated with MOG have no detectible pErk and delayed phosphorylation of c-Jun. Splenocytes from SMARTA or 2D2 mice were stimulated with 10 µM of the indicated antigen and signaling events were assessed. (A) A representative plot of pErk expression assessed at various time points by flow cytometry, gated on CD4^+^ cells. (B) A representative plot of p-c-Jun expression assessed at various time points by flow cytometry, gated on CD4^+^ cells. Graphical representation of averaged p-Erk (C) and p-c-Jun (D) expression are from at least three independent experiments at various time points. (E) Cell proliferation was assessed after treatment with the Erk-specific MEK inhibitor U0126. All experiments were repeated at least three times.

Erk plays a key role in positive T cell signaling events [38], [39], although it has been noted that in human cells Erk signaling can contribute to limitation of naïve T cell activation [40]. To explore the impact that TCR affinity has on T cell activation, phosphorylation of Erk in the MAP kinase pathway was analyzed. Phosphorylation events were visualized by flow cytometry to allow for detection and isolation of CD4^+^ T cells from other cell types [41]. Upon activation with gp61, SMARTA CD4^+^ T cells showed rapid phosphorylation of Erk1/2 by 5 min, peaking at 30 min, and remaining phosphorylated through 6 h (Figure 3a and c). Conversely, stimulation of 2D2 CD4^+^ T cells with MOG showed no appreciable accumulation of pErk at any time, from 5 min through 24 hours (Figure 3a and c). Activation of 2D2 CD4^+^ T cells with the higher 2D affinity NFM ligand resulted in up-regulation of pErk by 5 to 15 min, peaking at 60 min before slowly declining by 24 h (Figure 3a and c). The NFM response confirms that the 2D2 cells are not intrinsically deficient in their signaling capacity. This data highlights that, despite proliferative and cytokine responses (Figure 2), there is a dramatic difference in the initial signaling program of high (SMARTA and NFM) versus low (MOG) affinity agonists.

Although there is no notable accumulation of phosphorylated Erk upon 2D2 TCR ligation with MOG, T cell stimulation with low affinity ligands can allow highly reversible events, such as phosphorylation of Erk, to revert quickly back to the basal state, thus precluding detection by biochemical methods [42]. To determine if Erk phosphorylation plays a role in 2D2 T cell activation with MOG antigen, we assessed proliferation in the presence of the MEK1/2 inhibitor, U0126. At 10 µM, this inhibitor specifically targets MEK1/2 to inhibit the activation of Erk1/2 without acting on p38 MAPK, JNK, protein kinase C, or other pathways [43]. Treating 2D2 T cells with 10 µM U0126 prior to MOG stimulation resulted in a marked decrease in the amount of 2D2 proliferation (Figure 3e). Despite the lack of detectable pErk in 2D2:MOG cells, these results demonstrate that this pathway is nonetheless utilized for activation.

Another mediator of T cell activation, c-Jun, is an important target of Erk and the MAPK intermediate, JNK [44]–[46]. To further explore the signaling profile during low potency peptide interactions in self-reactive T cells, we assessed the kinetics of c-Jun phosphorylation [42], [47]. With either SMARTA:gp61 or 2D2 triggered by NFM, c-Jun is phosphorylated within 1 hour, while phosphorylated c-Jun (p-c-Jun) is not detected in 2D2 CD4^+^ T cells stimulated with MOG until 3–6 hours later (Figure 3b and d). Interestingly, the magnitude of the response also correlates with the effective 2D affinity of the ligands, as more cells express both pErk and p-c-Jun in SMARTA:gp61, followed by 2D2:NFM and finally 2D2:MOG.

**Figure 4:**
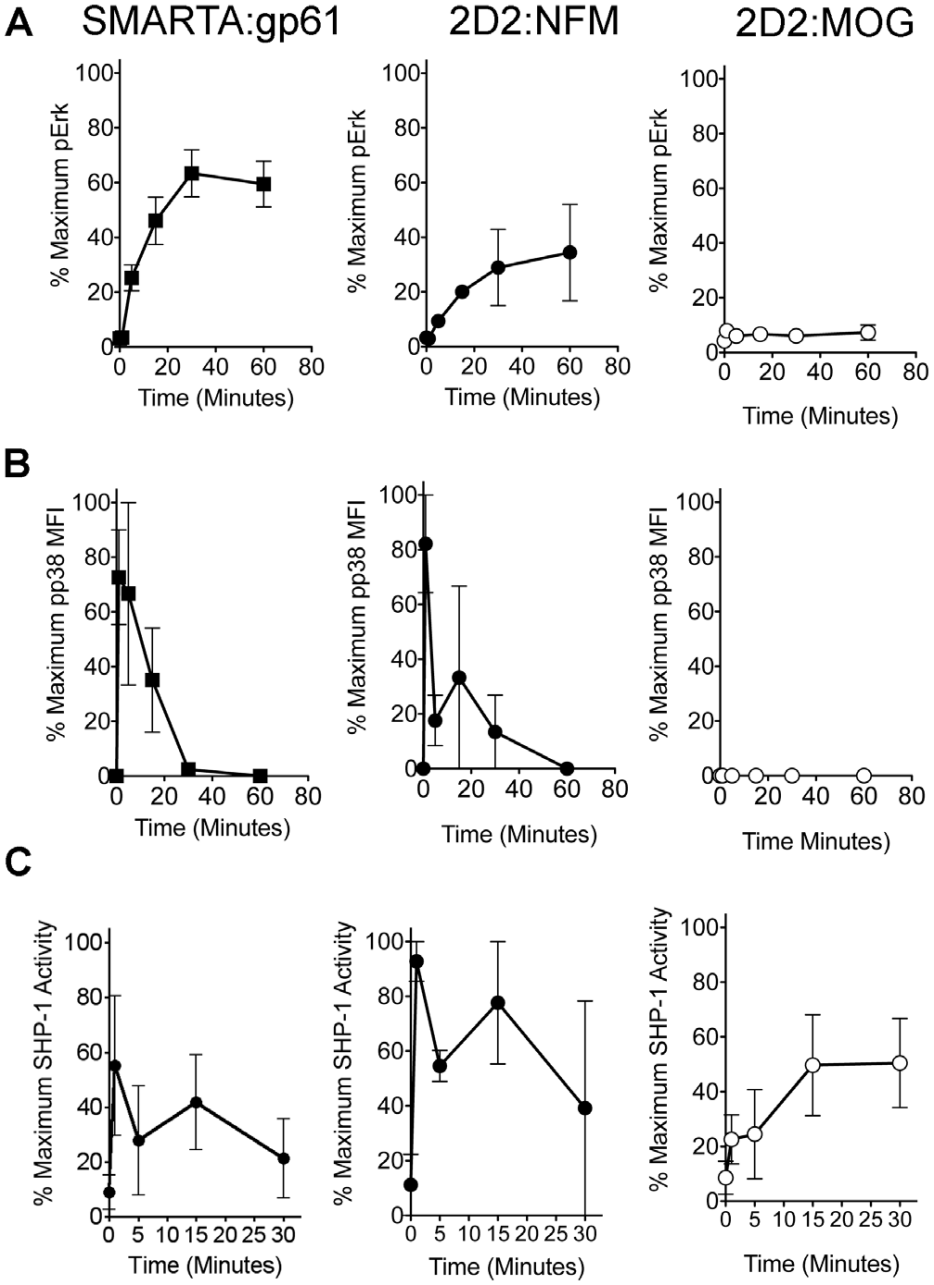
2D2 T cells stimulated with MOG have an altered signaling program. (A) Splenocytes from SMARTA or 2D2 mice were stimulated with 10 µM of the indicated antigen and the percent of maximal Erk phosphorylation was assessed at various time points by flow cytometry (gated on CD4^+^ cells). (B) Splenocytes from SMARTA or 2D2 mice were stimulated with 10 µM of the indicated antigen and the percent of maximal p38 MAPK phosphorylation was assessed at various time points by flow cytometry (gated on CD4^+^ cells). (C) Splenocytes from SMARTA or 2D2 mice were stimulated with 10 µM of the indicated antigen and SHP-1 phosphatase activity was assessed at various time points using a colorimetric assay for free phosphate with a phosphorylated peptide substrate specific for SHP-1. The percent of maximal response was assessed to highlight the kinetics of the signaling response and was calculated using GraphPad Prism/ All experiments were repeated at least three times.

To further define the delayed signal transduction in T cells with a low affinity for antigen, we assessed the kinetics of p38 MAPK phosphorylation, which can influence differentiation of Th1 cells, IFN-γ production and possibly proliferation [48], [49]. Analogous to the phosphorylation kinetics of Erk, phosphorylation of p38 MAPK was similar in SMARTA:gp61 and 2D2:NFM but absent in 2D2:MOG (Figure 4a and b). The percent of maximal phosphorylation of either Erk or p38 is shown to compare the kinetics of the response for each positive signaling mediator. For both SMARTA:gp61 and 2D2:NFM, phosphorylation of p38 peaked by 5 minutes and declined to baseline by 30 to 60 minutes (Figure 4b) while phosphorylation of Erk peaked by 30 to 60 minutes before declining (Figure 4a and Figure 3a and c). However, with 2D2:MOG, there is no apparent accumulation of phosphorylated p38 throughout the time course (Figure 4b), similar to the lack of detectable pErk accumulation (Figure 3 and 4a).

Previously, we have shown that the negative regulator SHP-1 plays a role in controlling an autoimmune response to MOG [50], and in CD8+ T cells that the peak of SHP-1 activity occurs at 1 min in response to antigen [51]. Here, the kinetics of SHP-1 activity was analyzed following T cell activation. The phosphatase assay employed allows for determination of the amount of free phosphate released by immunoprecipitated SHP-1 from cell lysates using a SHP-1 specific phosphorylated substrate. Both SMARTA:gp61 and 2D2:NFM have up-regulated SHP-1 activity at 1 minute (Figure 4c). Strikingly, the peak of SHP-1 activity was delayed in 2D2 MOG stimulation (15 minutes) as compared to SMARTA:gp61 and 2D2:NFM (Figure 4c). In addition to the delay in peak activation, SHP-1 remains active in 2D2 MOG stimulation throughout the 30-minute time course. This data shows that multiple detectable signaling pathways are delayed or undetectable in 2D2:MOG activation, including both positive and negative feedback loops.

### Expression of activation markers in low affinity CD4+ T cells

**Figure 5:**
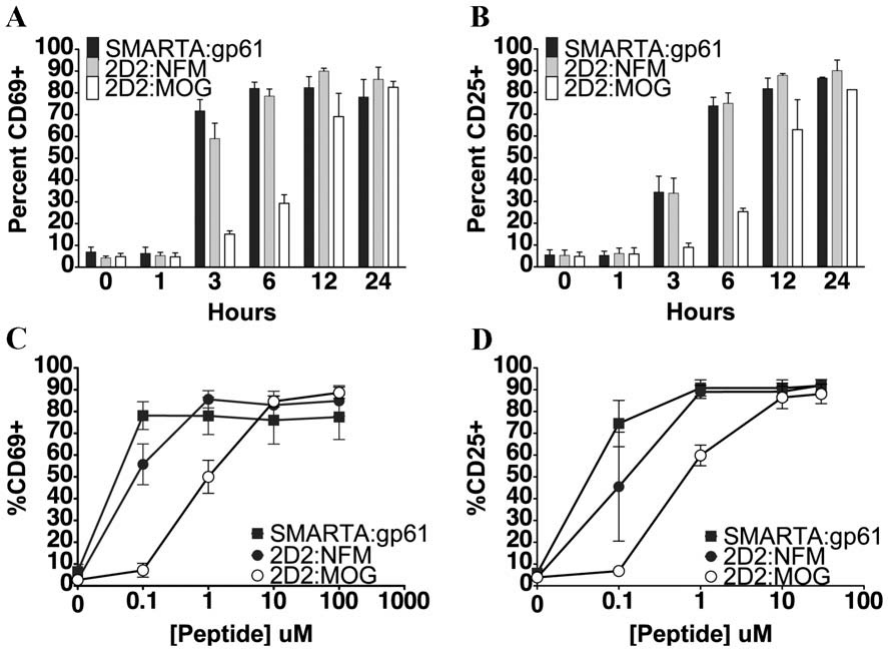
2D2 CD4^+^ T cell stimulation with MOG results in delayed expression of activation markers. SMARTA or 2D2 splenocytes were stimulated with 10 µM antigen for various time points and CD69 (A) and CD25 (B) expression on CD4^+^ cells was analyzed by flow cytometry. SMARTA or 2D2 splenocytes were stimulated with various concentrations of antigen for 24 hrs. and CD69 (C) and CD25 (D) expression on CD4^+^ cells was analyzed by flow cytometry. Experiments were performed at least three times.

This delay with signaling events could eventually translate to delays in downstream responses. We analyzed the expression of CD69, a T cell activation marker downstream of the Ras/Erk MAP kinase pathway [52] by flow cytometry and found that 2D2 T cells stimulated with MOG showed a delay in CD69 up-regulation (Figure 5a). Both SMARTA:gp61 and 2D2:NFM stimulation resulted in complete up-regulation of CD69 by 4 hours whereas stimulation with MOG did not completely up-regulate CD69 until 24 hours (Figure 5a). The magnitude of the CD69 response was comparable at high doses of peptide at 24 hours with each peptide, but 2D2:MOG needed 10- to 100- fold more antigen for maximal response (Figure 5c).

Similarly, the high affinity IL-2 receptor and marker of T cell activation, CD25, was completely up regulated by 24 hours for all peptides, yet MOG displayed a significant delay (Figure 5b and d). In SMARTA cells, activation with gp_61–80_ resulted in peak CD25 expression by 6 hours (Figure 5b). This was similar to the kinetics of CD25 expression in 2D2 cells stimulated with NFM; however, 2D2:MOG did not attain peak expression of CD25 until much later (24 hours) (Figure 5b). Analogous to the delay in MAP kinase signaling, up-regulation of activation markers was delayed in 2D2:MOG as compared to 2D2:NFM or SMARTA:gp61.

### Delayed initiation of proliferation in low affinity CD4^+^ T cells

**Figure 6:**
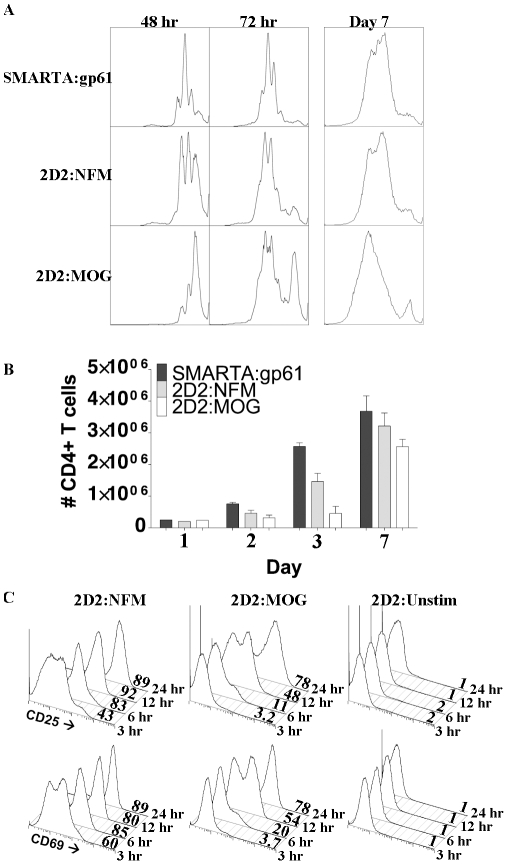
MOG stimulation results in delayed initiation of proliferation but eventual accumulation of CD4^+^ T cells. Splenocytes from SMARTA or 2D2 mice were CFSE labeled and stimulated with 10 µM of the indicated antigen for various times. (A) Representative plots from at least three independent experiments show CFSE dilution of CD4^+^ cells, assessed by flow cytometry, to detect proliferation at indicated times. (B) On various days, total CD4^+^ T cell numbers were assessed using BD Trucount tubes (BD Biosciences) to gauge cellular accumulation after peptide stimulation (On Day 7, SMARTA:gp61 and 2D2:NFM, p = 0.1; SMARTA:gp61 and 2D2:MOG p = 0.5; 2D2:NFM and 2D2:MOG p = 0.2). (C) After the indicated APC contact time, CD4+ T cells were MACS purified to remove the cells from the APCs. The percent of CD25+ and CD69+ CD4+ T cells was assessed by flow cytometry at 24 hours, as indicated on the x-axis. Experiments were performed at least three times; p values were generated using student's t-test on GraphPad Prism.

To extend our understanding of how low affinity interactions may be time-dependent, the kinetics of cellular proliferation were analyzed by CFSE dilution assay. In this assay there was a delay in proliferation in 2D2 T cells with MOG as compared to either SMARTA:gp61 or 2D2:NFM (Figure 6a). At 48 hours, the majority of SMARTA:gp61 and 2D2:NFM CD4^+^ T cells had entered cycle and undergone between one and two divisions; however, most of the CD4^+^ T cells in MOG activation remained undivided (Figure 6a). By 72 hours, all of the cells that entered cycle underwent approximately the same number of divisions regardless of the initiating peptide (Figure 6a). By day 3, there was a noticeable increase over baseline in the cell numbers of both SMARTA:gp61 and 2D2:NFM but not in 2D2:MOG, probably due to the increased fraction of cells entering cycle (Figure 6b). Interestingly, the total number of live CD4^+^ T cells after 7 days in culture was similar, in that SMARTA:gp61 (3.7×10^6^±4.9×10^5^ cells) and 2D2:NFM (3.2×10^6^±4.1×10^5^ cells) have a slightly greater, though not statistically significant, number of cells than 2D2:MOG (2. 6×10^6^±2.3×10^5^ cells) (Figure 6b). These data demonstrate that although the initiation of cellular proliferation is delayed in 2D2 T cells stimulated with MOG, the cells that do eventually enter the proliferative cycle retain the ability to divide and can eventually reach similar cell numbers of higher affinity TCRs.

Cell division in T cells has been shown to be programmed on presentation of antigen by APCs, requiring as little as a few hours to trigger a response [53]–[55]. Stimulation of 2D2 cells with NFM, but not MOG, allows for complete up-regulation of CD69 and CD25 by approximately 6 hours, whereas both peptides up-regulate activation markers by 24 hours (Figure 5a and b). To explore the importance of the time of antigen presentation as it relates to the apparent delay in MOG:2D2, the extent of activation was examined at 24 hours after various periods of T cell to APC contact time. CD4^+^ T cells were allowed 3, 6, 12 or 24 hours of contact time before analysis of CD69 and CD25 expression at 24 hrs. In 2D2:NFM, nearly half of the cells up-regulated CD69 and CD25 within 3 hours of APC contact time; however it took at least 12 hours for 2D2:MOG to up-regulate the activation markers to the same extent (Figure 6c). Within 6 to 12 hours of contact time, nearly all of NFM:2D2 CD4^+^ T cells up regulated CD69 and CD25. At 24 hours of APC contact time, both NFM and MOG allowed near complete activation of the cells (Figure 6c). This data shows that 2D2 T cells stimulated with a low affinity peptide need an extended period of time in contact with pMHC to reach maximal activation in comparison to higher affinity interactions.

## Discussion

It is generally assumed that auto-reactive T cells are of lower affinity than T cells specific for foreign antigen. Our lab has shown that in responses to a foreign antigen, gp61, or a self-peptide, MOG, the polyclonal T cell repertoire encompasses a similar wide breadth of affinities with the response to MOG including more low affinity T cell clones [10]. To further understand the relationship of affinity to T cell response, we examined viral specific and self-reactive TCR transgenic T cells (SMARTA and 2D2, respectively). We found that 2D2 T cells were of low affinity, compared to SMARTA T cells, and failed to be detected in the 2D micropipette assay or to react with a peptide-specific tetramer by flow cytometry. Surprisingly given their low affinity, we and many others have employed MOG-activated 2D2 T cells to induce EAE, indicating low TCR affinity for antigen does not preclude autoimmune disease [19], [22], [50], [56]–[58].

An alternative explanation for the lack of tetramer staining and 2D binding in 2D2 cells could relate to the findings that peptides presented by MHC class II, especially murine I-A^b^, can bind the MHC in different registers through the use of different MHC anchor residues [28]–[31]. Epitope mapping in 2D2 T cells revealed that the core encompasses MOG_39–48_, the epitope engineered in the MOG:I-A^b^ tetramer (Table I). Additionally, we have found that the MOG:I-A^b^ monomer identifies the majority (>70%) of the CD4^+^ T cells in the CNS at the peak of EAE [10]. The lack of tetramer staining and undetectable 2D affinity is unlikely due to an alternative register recognized by 2D2 T cells, but instead caused by the considerably lower affinity of this TCR to MOG.

The low affinity of 2D2 T cells for MOG led to a qualitative difference in the kinetics of T cell activation (Figure 3, 4, 5, 6), apparent as a delay in signaling, up-regulation of activation markers, and subsequent proliferation. However, analysis at later time points show that the T cells can compensate for this apparent early defect (Figure 6). This indicates that, under appropriate conditions that include continued access to antigen (Figure 6), time can offset low affinity for a ligand and allow for a slow accumulation of signaling intermediates that eventually lead to a complete response. This idea of qualitative as opposed to quantitative differences in peptide antigens as it relates to T cell responses has also been reported elsewhere and suggests that following CD4 TCR ligation, signaling pathways can diverge to allow for various functional outcomes of demonstrable TCR:antigen interactions [42], [59].

There has been much interest in determining the early signaling events in T cells after TCR ligation; however, the kinetics of the various signaling intermediaries involved and how timing affects the net outcome of signaling is still under investigation. The timing of signal propagation through both positive and negative mediators may play a key role in modulating T cell activation with low potency ligands. In this study we aimed to assess a few of the many potential differences in activation kinetics of a high affinity viral-specific T cell from SMARTA mice to a low affinity self-reactive T cell from 2D2 mice. Mathematical modeling of TCR signal transduction theorizes that positive and negative feedback loops activated during T cell interactions with antigen presenting cells allow for discrimination between a range of ligand affinities [39], [60]. In fact, one model proposes a balance between the positive signals driven by Erk activation and negative signals driven by SHP-1 to regulate T cell activation [39]. Here, we show that even with undetectable Erk or p38 phosphorylation and delayed, but sustained SHP-1 activity, self-reactive T cells that encounter low affinity ligands can still undergo full activation. This suggests that these feedback mechanisms may be more complicated than initially described and that a smoldering positive signal may be able to drive T cell activation under the appropriate conditions.

Importantly, in addition to differences in positive signaling, we show that there is delayed, but sustained up-regulation of SHP-1 activity in 2D2:MOG interactions, but not in NFM (or SMARTA:gp61) stimulation (Figure 4). We, and others, have shown an important role for SHP-1 in regulating responses to low potency antigens [50], [51], [61], [62]. Specifically, we have shown that an LCMV mutant epitope allowing for viral escape induces delayed SHP-1 activation in CD8+ T cells [51]. In these experiments, the quick burst of SHP-1 activity in agonist stimulation is not unexpected, as this denotes the initial burst of signaling initiated by a potent pMHC complex. The quick recovery of SHP-1 to an inactive state during strong peptide interactions (SMARTA:gp61 and 2D2:NFM) supports the licensing of that cell to undergo activation and proliferation. The delayed but sustained activation of SHP-1 following MOG stimulation indicates an altered signaling profile in these cells and corresponds with the lower functional avidity of 2D2 T cells for MOG. We suggest that the interplay between delayed or negligible levels of positive signaling (c-Jun or Erk and p38, respectively) and the sustained negative activity of SHP-1 synergistically affects the outcome of T cell signaling in low affinity TCR interactions; however, given adequate time cells are able to overcome this delay in activation to divide and survive.

The absence of measured MOG reactivity using specific tetramer or the micropipette analysis may very well indicate the importance of time for these affinity measures. Delayed on-rates or very rapid off-rates could affect the ultimate outcome of T cell stimulation. However, with the current assays it is difficult to distinguish these possibilities. Peptide:MHC tetramers work by increasing the avidity of the TCR:pMHC by allowing an improved chance of interaction between TCR and the multimeric structure [63], [64], which would be dependent on the binding kinetics [10]. Time is similarly important for the micropipette measurements as low affinity translates to a low binding probability [5], [10]. Longer antigen exposure time equates to a larger total number of TCR:pMHC encounters, hence a larger cumulative number of TCR bonds, which increases the binding probability [65]. Previously, we have shown that 2D2 T cells require high expression levels of TCR for response, supporting a requirement of an increased number of TCR bonds [21]. Thus, the persistent exposure of an autoimmune antigen may be able to compensate for its low affinity to yield a cumulative number of TCR bonds sufficient for T cell activation.

We have reported that the average TCR affinity for a polyclonal MOG-specific population is on the order of 10^−5^ µm^4^ [10]. This implies that 2D2 cells represent the lowest affinity T cells in the spectrum of polyclonal TCR affinities during an autoimmune response. When compared to the CD8+ OT-I system, the 2D2 affinity for MOG is even below the level reported for the ovalbumin TCR antagonists [5]. This indicates that 2D2 T cells are of very low affinity and raises the issue of how T cells with such low affinity are relevant to T cell responses. Normally, during an acute infection the foreign antigen is transiently expressed for a limited amount of time [11], [66]. Self-antigens on the other hand are constantly available to be presented to T cells, potentially extending the length of time for triggering of the T cell [15], [16]. Taken together, this data implies that analysis of T cell activation over a short time frame may not allow for a full understanding of agonistic properties of antigens, specifically in autoreactive T cells that are able to encounter low affinity peptides over extended periods of time.

Interestingly, the importance of time and the availability of antigen in the ability of T cells to reach the thresholds for signaling events highlights the fact that autoreactive T cells may be able to use time, through either sustained or short repeated engagements, to achieve a response [67]–[69]. Upon multiple instances of stimulation, high affinity T cells undergo exhaustion as one method to limit damage to the host in response to ineffective clearance of an infection [70], [71]. Potentially, the smoldering T cell response observed during autoimmune disease may result, at least in part, from the activation of very low affinity T cells that can escape tolerance mechanisms given sufficient time and access to self-antigens.

## Materials and Methods

### Transgenic Mice

This study was performed in strict accordance with the recommendations in the Guide for the Care and Use of Laboratory Animals of the National Institutes of Health. MOG35-55 specific TCR transgenic 2D2 mice (Jackson Labs, C57BL/6-Tg(Tcra2D2,Tcrb2D2)1Kuch/J) and gp61–80 specific TCR transgenic SMARTA mice [17], [18] were bred, housed and used with specific approval from the Institutional Animal Care and Use Committee-approved protocol of the Emory University Department of Animal Resources facility (IUCAC Number: DAR-2000870-061414). All mice were used for experiments at 6–8 weeks of age.

### Peptides and Reagents

LCMV gp61–80 (GLNGPDIYKGVYQFKSVEFD) and mouse NFM15–35 (RRVTETRSSFSRVSGSPSSGF) and MOG35–55 (MEVGWYRSPFSRVVHLYRNGK) were synthesized in-house using F-moc chemistry on the Prelude peptide synthesizer (Protein Technologies). Culture medium consisted of RPMI 1640 medium (Mediatech) supplemented with 10% FBS (HyClone), 2 mM L-glutamine (Mediatech), 0.01 M HEPES buffer (Mediatech), 100 µg/ml gentamicin (Mediatech), and 2×10^−5^ M 2-ME (Sigma-Aldrich). Oxidation buffer consisted of 20 mM Tris-HCl (pH 7.5), 150 mM NaCl, 1 mM Na_2_EDTA, 0.5% Igepal, 1 mM Na_3_VO_4_, and 1/100 protease inhibitor cocktail I (Calbiochem) to inhibit degradation of cellular proteins following lysis of the cells.

### 2D TCR affinity analysis by micropipette adhesion frequency assay

Human red blood cells (RBCs) were isolated from healthy volunteers at the Georgia Institute of Technology in accordance with specific approval from the Georgia Institute of Technology Institutional Review Board (protocol number: H07343) and prepared as previously described [5]. In accordance with ethical guidelines, written and informed consent was obtained from all anonymous volunteers prior to blood collection. RBCs were coated with various concentrations of biotin-X-NHS (Calbiochem), followed by 0.5 mg/ml streptavidin (Pierce) and then 1–2 µg of pMHC II monomer. The MOG_38–49_:I-A^b^, NF-M_18–30_:I-A^b^, GP_66–77_:I-A^b^ monomers were provided by the NIAID Tetramer Core Facility at Emory University. The pMHC-coated RBCs were stained with anti-MHC II FITC Ab (M5/114.15.2; BioLegend) and T cells were stained with anti-TCRβ FITC Ab (H57–597; eBioscience). The site densities of I-A^b^ monomers per RBC and TCRs per T cell were derived using anti-FITC MHC II, anti-TCR antibodies, and FITC MESF beads (Bangs Labs) and normalized for the F/P ratios of the antibodies.

The details of the micropipette adhesion frequency assay have been described [5], [20]. Briefly, the adhesion was measured following contact of a single T cell and pMHC-coated RBC on opposing micropipettes. At the end of the contact time, the T cell was retracted and the presence of adhesion (indicating TCR:pMHC ligation) was observed microscopically by elongation of the RBC membrane. The adhesion frequency (*P_a_*) was calculated by performing the contact-retraction 50 times per T cell-RBC pair. A 5 second contact time was chosen in all experiments because the *P_a_* had reached equilibrium and remained constant despite further increase in contact time. The effective 2D affinity (*A_c_K_a_*) was calculated using the average *P_a_* according to the following equation:
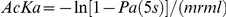
where *m_r_* and *m_l_* reflect the receptor (TCR) and ligand (pMHC) densities, respectively.

### T cell Tetramer Staining

As performed previously, splenocytes from SMARTA or 2D2 mice were incubated for 7 days at 37°C with either gp61–80 or MOG35–55, respectively [10]. Live, previously activated cells were isolated using a Ficoll gradient, washed, and stained for tetramer analysis. Live cells were incubated with 4 µg/ml MOG_38–49_:I-A^b^ (8–20 h) [27], GP_66–77_:I-A^b^ tetramers (3–4 h), or hCLIP_103–117_:I-A^b^ (NIAID Tetramer Core Facility at Emory University, Atlanta, GA) in complete RPMI at 37°C. The cells were washed with buffer containing 1× PBS, 0.1% BSA, and 0.05% sodium azide. Cells were then stained with anti-CD4-APC (RM4.5) (BD-Bioscience) and 7-AAD for 30 minutes on ice. The percentage of tetramer-PE positive cells was determined in live (7-AAD negative) CD4-positive populations. All flow cytometric analysis was performed on a FACSCalibur (BD) and data were analyzed using FlowJo (Tree Star).

### T cell Proliferation

For [^3^H]-thymidine uptake, 6×10^5^ naive splenocytes from 2D2 or SMARTA mice were incubated in a 96-well plate with the indicated concentration of peptide. In some assays (as indicated), cells were pretreated for 30 min with the MEK inhibitor U0126 (Promega) at 10 µM. After 48 h in culture, cells were labeled with 0.4 µCi/well [^3^H]-thymidine. After 18–24 h, the plates were harvested on a FilterMate harvester (Packard Instrument) and analyzed on a 1450 LSC Microbeta TriLux counter (PerkinElmer) [72].

For CFSE analysis, naïve splenocytes from either 2D2 or SMARTA mice were labeled with CFSE and 1.5×10^6^ cells were incubated in 24-well plates with 10 µM peptide for a given time period before being stained with CD4 APC and 7-AAD and analyzed on a FACSCalibur.

### T cell IL-2 ELISA

Splenocytes (1.5×10^6^) from 2D2 or SMARTA mice were incubated in a 24-well plate with the indicated concentration of peptide. After 24 h in culture, supernatants were removed and placed on microtiter plates coated with purified anti-IL-2 (5 µg/ml clone JES6-1A12; BD Pharmingen) overnight at 4°C. Recombinant IL-2 (BD Pharmingen) was used as a standard. Captured cytokines were detected using biotinylated anti-IL-2 (100 µg/ml JES6-5H4, 100 µl per well; BD Pharmingen) and detected using alkaline phosphatase-conjugated avidin (Sigma-Aldrich) and *p*-nitrophenyl phosphate substrate (Bio-Rad). Colorimetric change was measured at dual wavelengths of 405 and 630 nm on a Microplate Autoreader (Biotek Synergy HT) [72].

### Analysis of T cell signaling

For time courses that included short peptide stimulation (≤60 min), fibroblasts transfected with I-A^b^ (clone FT7.1C6) [73] were plated out in 24-well plates and incubated until confluent (24 h), pre-pulsed with the indicated dose of antigen for 1–2 h and washed. Naive splenocytes were run over a Ficoll gradient and 3×10^6^ cells were added to each well of pre-pulsed fibroblasts. Cells were spun at 600 rpm for 1 min to begin peptide stimulation and allowed to incubate at 37°C for the indicated time points. For time courses with only long peptide stimulation (≥60 min), naïve splenocytes (3×10^6^) were stimulated with the indicated dose of antigen for the duration of the time course.

For analysis of protein phosphorylation, cells were taken off fibroblasts at the indicated time points and approximately 300,000–500,000 splenocytes were stained for intracellular signaling events. Cells were fixed for 10 min with methanol free formaldehyde at room temperature and permeabilized with 100% ice-cold methanol for 10 min on ice. Cells were then stained with antibodies to CD4 (RM4-5, BD Biosciences), p-p44/42 (D13.14.4E, Cell Signaling), p-c-Jun (KM-1, Santa Cruz Biotechnology), and/or phospho-p38 MAPK (3D7, Cell Signaling) for 30 min on ice, washed, and immediately analyzed by flow cytometry. Flow cytometry was performed on a BD FACSCalibur and data were processed using FlowJo software (Tree Star). FACS wash consisted of PBS, 0.05% sodium azide, and 0.1% BSA.

For analysis of SHP-1 activity, cells were taken off pre-pulsed fibroblasts at the indicated time points, lysed in oxidation buffer and spun at 14,000 rpm for 5 min. SHP-1 was immunoprecipitated with 2 µg of anti-SHP-1 Ab (C19, Santa Cruz biotechnology) overnight, collected with protein A beads for 1 h, and protein A beads were washed once with oxidation buffer and twice with wash buffer (25 mM HEPES (pH 7.2), 50 mM NaCl, and 2.5 mM EDTA). SHP-1 substrate peptide (AEEEIpYGEFEA) was added at a final concentration of 1 mM in Tyr assay buffer with 5 mM DTT (Upstate Biotechnology) and incubated with immunoprecipitated SHP-1 for 1 h at 37°C. Released phosphate was detected by addition of malachite green (Upstate Biotechnology) [51], [74], [75].

### Analysis of Surface Markers

For continuous peptide stimulation, splenocytes (3×10^6^) from 2D2 or SMARTA mice were stimulated for the indicated time points in 24-well plates, washed in FACS buffer and surface stained for CD4 (RM4-5, BD Bioscience), CD25 (PC61, BD Bioscience), and CD69 (H1.2F3, BD Bioscience) for 30 min on ice. Cells were then washed, stored at 4°C and run on a flow cytometer within 24 hours.

For stimulation with various APC contact times, splenocytes (3×10^6^) from 2D2 mice were stimulated for the indicated time points in a 24-well plate prior to CD4^+^ MACS purification carried out as per manufacturer's instructions (CD4^+^ T Cell Isolation Kit, MACS Miltenyi Biotec). The isolated CD4^+^ T cells were then resuspended in R10, placed in a well in the 24-well plate and incubated for the remaining time in the 24 hour time course, to allow for further protein production and up-regulation after the limited stimulation time. At 24 hours, the remaining cells were also purified and all cells were stained on ice for 30 minutes with CD69 FITC, CD4 PE, 7AAD, CD25 APC and analyzed on a FACSCalibur flow cytometer.

### Statistical Analysis

All data analysis was performed on GraphPad Prism (Software for Science).
